# Graphyne-3: a highly efficient candidate for separation of small gas molecules from gaseous mixtures

**DOI:** 10.1038/s41598-021-95304-z

**Published:** 2021-08-11

**Authors:** Khatereh Azizi, S. Mehdi Vaez Allaei, Arman Fathizadeh, Ali Sadeghi, Muhammad Sahimi

**Affiliations:** 1 Department of Physics, University of Tehran, Tehran, 14395-547 Iran; 2 School of Physics, Institute for Research in Fundamental Sciences (IPM), Tehran, 19395-5531 Iran; 3 Institute for Computational Engineering and Sciences, University of Texas at Austin, Austin, TX 78712 USA; 4 Department of Physics, Shahid Beheshti University, Tehran, Iran; 5 School of Nano Science, Institute for Research in Fundamental Sciences (IPM), Tehran, 19395-5531 Iran; 6 Mork Family Department of Chemical Engineering and Materials Science, University of Southern California, Los Angeles, CA 90089-1211 USA

**Keywords:** Materials science, Nanoscience and technology

## Abstract

Two-dimensional nanosheets, such as the general family of graphenes have attracted considerable attention over the past decade, due to their excellent thermal, mechanical, and electrical properties. We report on the result of a study of separation of gaseous mixtures by a model graphyne-3 membrane, using extensive molecular dynamics simulations and density functional theory. Four binary and one ternary mixtures of H$_2$, CO$_2$, CH$_4$ and C$_2$H$_6$ were studied. The results indicate the excellence of graphyne-3 for separation of small gas molecules from the mixtures. In particular, the H$_2$ permeance through the membrane is on the order of $10^7$ gas permeation unit, by far much larger than those in other membranes, and in particular in graphene. To gain deeper insights into the phenomenon, we also computed the density profiles and the residence times of the gases near the graphyne-3 surface, as well as their interaction energies with the membrane. The results indicate clearly the tendency of H$_2$ to pass through the membrane at high rates, leaving behind C$_2$H$_6$ and larger molecules on the surface. In addition, the possibility of chemisorption is clearly ruled out. These results, together with the very good mechanical properties of graphyne-3, confirm that it is an excellent candidate for separating small gas molecules from gaseous mixtures, hence opening the way for its industrial use.

## Introduction

Separation of gaseous mixtures into their constituent components is a problem of fundamental scientific and industrial importance. For example, separation (and subsequent sequestration) of CO$_2$, the prime culprit in the green house effect and the resulting climate change, from many gaseous mixtures, and in particular those that contain hydrocarbons, is of much current interest. Another example is increased recovery of oil, for which one method is based on injecting CO$_2$ into oil reservoirs. A third important problem is separation of hydrogen from gaseous mixtures. Growing interest in the hydrogen economy has motivated research on fabrication of inorganic, hydrogen-permselective membranes for use in the processes related to H$_2$ production and its separation from other reaction products that take place at high temperatures and pressures. It is due to such important practical problems, as well as many others, that the development of materials for efficient separation of gaseous mixtures into their individual components remains a problem of much current interest.

An important class of porous materials consists of nanoporous membranes that have been under active investigations, both experimentally and by computer simulations, for separation of gaseous mixtures. Many research groups have fabricated a variety of amorphous nanoporous membranes for separation of gaseous mixtures, including carbon molecular-sieve^1^, silicon-carbide^2^ and polymeric membranes^3^. Each type of the membranes has its own advantages and disadvantages. For example, polymeric membranes, while efficient at low temperatures, are not suited for high-temperature applications. Thus, despite considerable success, the search for membranes and materials with optimal performance, and in particular selectivity of one gas over other gases, as well as cost effectiveness has continued.

A high-performance membrane must have a few characteristics. One is its ability to allow large fluxes of the species of interest, for separation to pass through. For pressure-driven membranes, the flux is proportional to the permeance and inversely related to the pressure difference. The permeance itself is estimated by the ratio of the permeability and the thickness of the membrane, and is the prime quantity considered for comparing membranes. Thus, a high performance membrane must be as thin as possible. More information can be found elsewhere^4^. However, the thin material must also be able to resist mechanical failure and cracking. Otherwise, it will break under the action of large external pressure gradients that are typically applied to many membranes in order to facilitate flow and transport through them.

Since the fabrication of graphene by Novoselov et al.^5^ and the 2010 Physics Nobel Prize for their achievement, graphene and its allotropes have attracted wide attentions due to their potential applications. Being mechanically robust, having a two-dimensional (2D) structure that implies minimum thickness, and special electrical and thermal properties make the graphene family of materials a potential candidate for novel applications^6,7^. Gas separation is one such application of graphene as a membrane^8,9^. They have been studied for separation of hydrogen isotopes^10–13^, helium^14^, and its isotopes, $^3$He/$^4$He^15^, binary mixtures of CO$_2$ and N$_2$^16,17^, and of H$_2$ and N$_2$^18^, as well as separation of other types of gaseous mixtures^19^. In addition, graphene has been proposed as filter and membrane for water purification and desalination^20–25^; for reviews of the application of gas separation and water desalination using graphene and graphene oxide see Refs.^26–28^.

Graphyne, another 2D crystalline carbon allotrope, also has a thickness of one layer of carbon but is built from both triple- and double-bonded units of two sp- and sp$^2$-hybridized carbon atoms^29^. Graphyne is endowed by the usual intrinsic properties of carbonenous materials, such as excellent chemical stability, large surface area, and electronic conductivity. Most importantly, and from a practical view point, graphyne has high potential for various application because it can be synthesized^30–40^.

Given the structure of graphyne-3 with its intrinsic pores that are of the same size as those of small molecules, it can be a prime candidate for separation of gaseous mixtures that contain small gas molecules. Qiu et al.^41^   reviewed the potential of graphyne families as a membrane for gas separation and water desalination. The most commonly studied membrane for gas separation is graphdyne, while graphyne-3 has only been studied for CO$_2$ separation from N$_2$^17^ and H$_2$, and H$_2$O^18^.

In this paper we report on the results of a study by extensive molecular dynamics (MD) and density functional theory (DFT) simulations of the potential of graphyne-3 as a membrane for separation of several gaseous mixtures containing CH$_4$, C$_2$H$_6$, CO$_2$ and H$_2$. Such mixtures are of industrial importance, and their separation is of much curren﻿t interest. We report on the results of the MD simulations for studying the separation of four binary and one ternary gas mixtures. The effect of the pressure difference and the composition of the mixtures on the separation is also studied. Moreover, we also study the effect of membrane rigidity on the separation processes, which is usually ignored in other studies. In addition, investigating the density profiles and residence times of the molecules near the membrane’s surface using MD and the averaged molecule-membrane interaction energy using DFT method indicates that small gases permeate through graphyne-3 sheets rather readily, and CO$_2$ and CH$_4$ have nearly similar behavior, whereas C$_2$H$_6$ faces a large barrier for passing through the membrane. We also report on the results of a series of DFT calculations aimed at verifying the possibility of any chemical reaction of the gases with graphyne-3. The result of our *ab initio* calculations excludes such chemical processes. All in all, our study points to graphyne-3 as an excellent medium for hydrogen and ethane separation, much better than the much more studied membranes made of graphene, and other types of materials. In particular, we demonstrate that graphyne-3 has superior properties and potential over those membranes that have so far been investigated for separating H$_2$ from a gaseous mixture.

The rest of this paper is organized as follows. In the next section we describe the details of MD and DFT simulations and explain the computational procedure. The results are presented and discussed in "Results and discussion" section. The paper is summarized in "Summary" section.

## Details of the computations

We first describe the details of the computations, including the MD simulations, the molecular models of the material and the gases, and the DFT calculations.

### MD simulations

**Figure 1:**
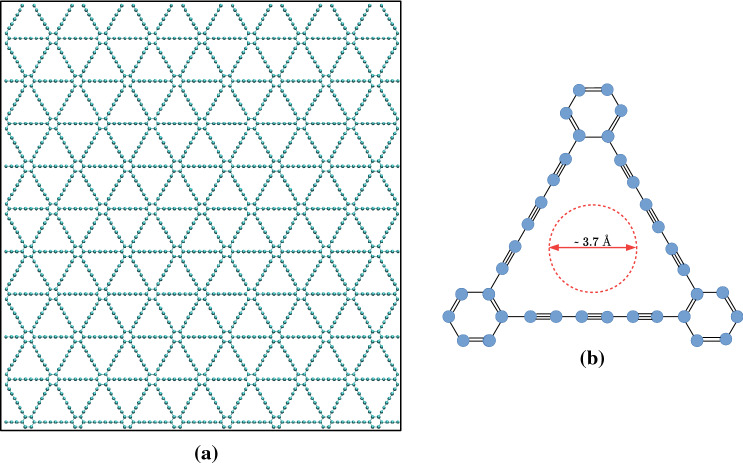
Structure of Graphyne-3. (**a**) The structure of the 2D sheet used as a membrane for gas separation. (**b**) The zoom-in structure, showing the atomic configuration. Blue dots are carbon atoms. The radius of the inscribed circle (pore radius) is about 3.7 Å.

The structure of graphyne-3, a 2D sheet of carbon atoms, is presented in Fig. 1a. Figure 1b shows the atomic configuration, showing the benzene-like rings connected by 6 carbon atoms, creating ideal-sized pores, with a radius of about 3.7 Å, making an efficient membrane for small gas separation.The gases studied here are H$_2$, CO$_2$, CH$_4$, and C$_2$H$_6$, which have kinetic diameters of 2.9 Å^42^, 3.3 Å^42^, 3.8 Å^42^, and 4.4 Å^43^, respectively, comparable to the pore size of graphyne-3. These are the mixture that one encounters in natural gas, as well as in many industrial applications. See, for example^44^. In some cases, water vapor is also present, but that is the subject of a future paper.

As the first step, the atomistic structure of graphyne-3 was generated, with the size of the sheet being $105.9\times 97.8\;\text {\AA }^2$. The structure was then relaxed by MD simulation in the *NPT* ensemble at 300 K and zero pressure, which generated a stress-free sheet, in order to obtain the equilibrium structure and the correct bond lengths. Then, three series of MD simulations and DFT calculations were carried out, as will be discussed in detail shortly.

**Figure 2:**
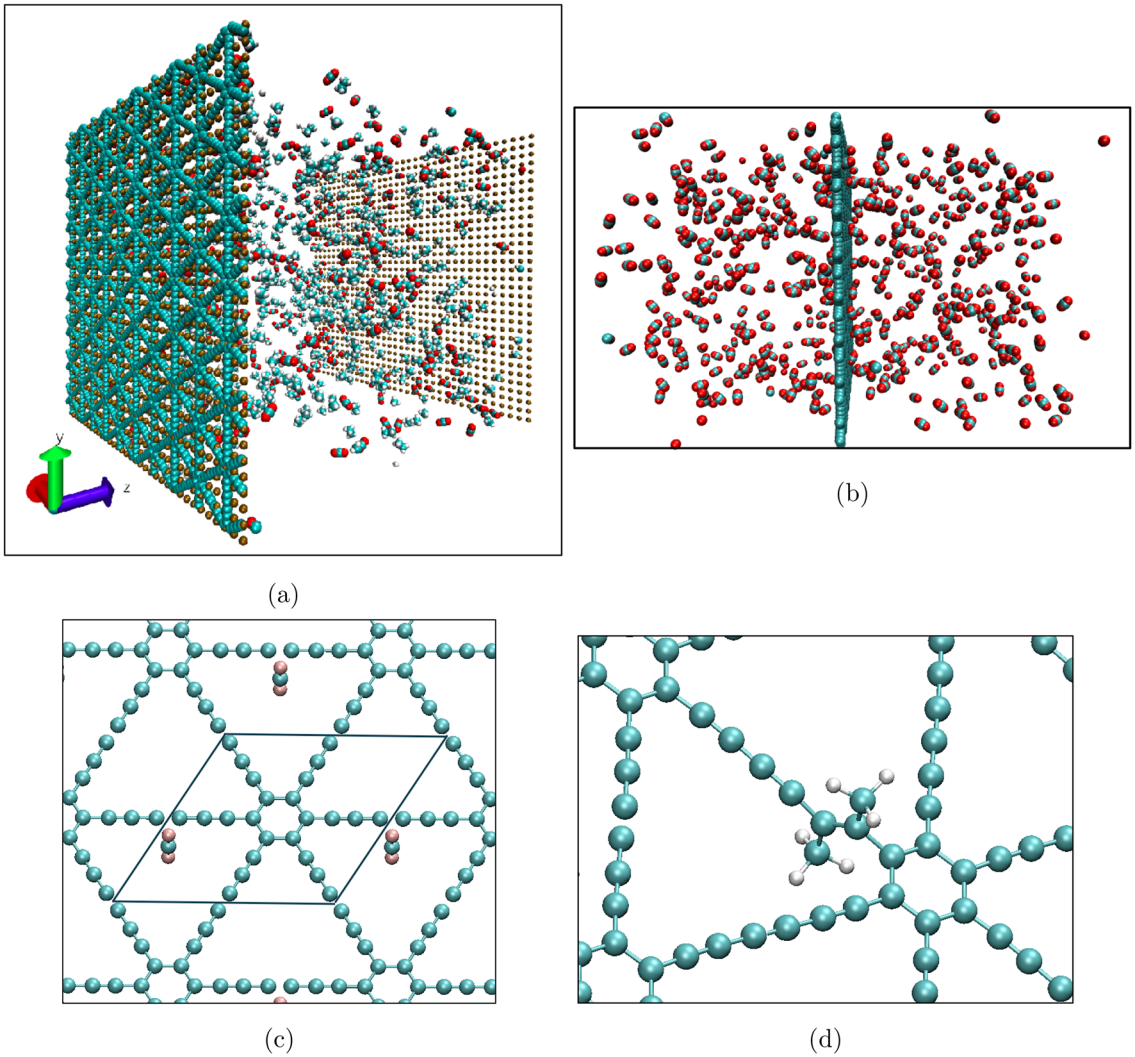
Simulations setup, with (**a**) and (**b**) being for MD simulations. (**a**) Series one and two, studying the separation of different mixtures at constant pressure (series 1), and the effect of pressure, mixture concentration, and system size in CH$_4$/CO$_2$ separation (series 2). (**b**) The setup for the third series of MD simulation to determine the density profile and residence time of each molecule type. (**c**) Unit cell (black box) used in the periodic DFT calculations for determining the interaction energy between the molecule CO$_2$ in *z*-direction, and the graphyne-3 sheet. (**d**) Chemical adsorption location determined by DFT calculations where C$_2$H$_6$ needs to be dissociated into two parts before developing chemical bonds with the sheet.

We first studied the separation of four binary mixtures, namely, CH$_4$/CO$_2$, CH$_4$/H$_2$, CH$_4$/C$_2$H$_6$, CO$_2$/H$_2$, and one ternary mixture of CH$_4$/CO$_2$/C$_2$H$_6$. To simulate equimolar mixtures, 330 molecules of each gas were placed between a piston and the membrane; see Fig. 2a. The MD simulations were carried out in three steps. First, the gas mixtures were equilibrated at 300 K, during which the membrane and the piston were held fixed at their positions. In addition, an auxiliary wall, as shown in the figure, was placed on the membrane to prevent gas transport through it. Duration of the first step was 500 ps. In the second step, also for 500 ps, the piston began to move smoothly in such a way that a constant pressure of about 30 bar was exerted by piston on gases. The auxiliary wall was still in place, so that the system could reach isothermal-isobaric equilibrium. In the final step the auxiliary wall was removed, so that the gases could pass through the graphyne-3 sheet. The size $L_z$ of the simulation box in the *z* direction, perpendicular to the graphyne-3 plane, was large enough, 300 Å, to make the pressure on the permeate side nearly zero. The duration of the third step was 1 ns. The simulations were carried out for two cases, one with rigid sheets, and a second series with deformable one, as described below, in order to study the effect of the sheet’s deformation on the separation properties of graphyne-3 for various gaseous mixtures.

In the second series of MD simulations, with the same setup as the first series, we studied the effect on gas permeation of the pressure and the composition of the mixtures, namely, the concentration of each molecule in the mixture, and the total number of molecules. As a typical binary mixture we selected CH$_4$/CO$_2$ system for this part of the study, since its separation is of prime importance^45^. The pressure difference was varied from about 20 to 120 bar. We also varied the CO$_2$ number fraction at a fixed pressure of about 30 bar in order to study the effect of the mixture’s composition, when the graphyne-3 sheets were deformable.

To quantify the behaviour of the gases near graphyne-3 sheets, a third series of simulation were carried out. We studied the self-diffusion of the gases at zero pressure gradient; See Fig. 2b, and computed the density profiles and the residence times of the gases near the graphyne sheet. The simulations were carried out for 5 ns after 500 ps of simulation for reaching equilibration. All the MD simulations were carried out using the LAMMPS package^46^, and a sample input file is available in the Supplementary Information.

### Molecular models and force fields

As mentioned earlier, we simulated two distinct cases. In one case the graphyne-3 nanosheet was assumed to be rigid, whereas in the second case it could be deformed as a result of the environment around it and the pressure gradient applied to the system. In the former case the carbon atoms were held fixed at their initial positions. To simulate the deformable case we utilized the AIREBO potential^47^ to describe the behavior of the membrane during the simulations.

CO$_2$ was modeled by the elementary physical model 2 (EPM2) by which it is represented as a three-site molecule with Lennard-Jones (LJ) interactions and partial point charges placed at each site’s center^48,49^. CH$_4$ and H$_2$ were represented by their full atomistic structures with the parameters given by Bucior et al.^48^ and Jorgenson^50^, while C$_2$H$_6$ was modeled by the OPLS-AA force field, with its parameters provided by Jorgenson^51^. To mention that using LJ is standard in this type of computations. If one looks at the literature, one finds a large number of examples with the same assumption. The reason is that it has been shown to be accurate, even in quantitative comparison between the simulations and data. See, for example, Xu et al.^52^. Of course, if there is any better potential for describing the interaction, it can be used. But, at this stage, our study is exploratory. All the intra-molecular parameters are summarized in Table S1 in the Supplementary Information. The interactions between gas and graphyne, between the gases, and between the gases and the piston were computed by the Lorentz-Berthelot mixing rule in which the LJ parameters of graphyne and the piston were assumed to be the same and were taken from Girifalco et al.^53^. No interaction was assumed between the graphyne and the piston. Table 1 lists all the LJ parameters and partial charges for the various atoms. We point out that, in principle, one should consider the possibility of reactions between the carbon atoms of graphyne-3 and the gases. In the next section we describe the DFT calculations utilized for our investigation on this purpose.

**Table 1:** Lennard-Jones radius ($\sigma$) and energy ($\epsilon$) parameters, and partial charges (*q*) of the individual atoms in molecular dynamics simulation.

Atom	$\epsilon$ (eV)	$\sigma$ (Å)	*q* (e)
C (CH$_4$)	0.00284	3.5	$-$ 0.24
H (CH$_4$)	0.0026	2.96	0.06
C (C$_2$H$_6$)	0.00284	3.5	$-$ 0.18
H (C$_2$H$_6$)	0.00129	2.5	0.06
C (CO$_2$)	0.006869	3.033	0.651
O (CO$_2$)	0.0024	2.757	$-$ 0.3255
H (H$_2$)	0.0026	2.96	0.0
C (graphyne)	0.0024	3.4	0.0
C (walls)	0.0024	3.4	0.0

### DFT calculations

The classical force fields used in the MD simulations are not designed to capture possible chemical reactions between graphyne-3 and the gas molecules. Thus, to investigate the validity of using the classical MD simulations for studying graphyne-3 as a membrane for gas separation, we benchmarked them against two series of reference DFT calculations. The DFT calculations were carried out within the generalized gradient approximation as implemented in the Quantum ESPRESSO package^54–56^. To model the effect of the core electrons, we used pseudopotentials of type USPP indicated as pbe-n-rrkjus_psl^57,58^. Plane waves of energies up to 40 Ry were used to expand the valance electrons wave function, which was converged to within $10^{-6}$ Ry, and four times this for the charge density. The hexagonal lattice of the pristine graphyne-3 sheet was found to have a unitcell of length of 11.98 Å.

A vacuum layer of height 20 Å was considered between the periodic images of the nano sheet in the normal-to-plane direction in all the calculations, in order to minimize the interactions between the spurious images. A $5\times 5 \times 1$ k-grid centered at the $\Gamma$-point was used to sample the first Brillouin zone for calculating the adsorption energies, while the positions of the atomic cores were relaxed to reach forces smaller than $2\times 10^{-4}$ Ry/bohr. To determine the potential energy landscape of the molecules close to the membrane, we carried out a series of calculations for several positions of the molecule from the membrane. The molecule and membrane are kept frozen and the interaction energy as a function of their relative position is calculated. The DFT-D2 method of Grimme^59^ was used to take into account the van der Waals interactions between the molecule and the membrane.

## Results and discussion

In this section we present the results of the study of gas separation through graphyne-3. As discussed earlier, permeation of the various mixtures, and the effect of the pressure and concentration on the permeation have been investigated. The density profiles and residence times, determined by MD simulations, as well as studying any possible reactions and the interaction energy, calculated by the DFT studies, help us to understand the permeation properties.

### Permeance of the gases and selectivity of graphyne-3

We first present the results for transport of the gas molecules in the five mixtures, namely, CH$_4$/CO$_2$, CH$_4$/H$_2$, CH$_4$/C$_2$H$_6$, CO$_2$/H$_2$, and CH$_4$/CO$_2$/C$_2$H$_6$, and the permeation and selectivity of the gases passing through both the rigid and flexible graphyne-3 membrane. As explained earlier, after equilibrium was reached at the end of the third step of the MD simulations, we computed the time-dependence of the number of gas molecules passing through the membrane, and normalized it by the area of the membrane in order to compute the number fluxes for all the gases in the mixtures. The ratio of the flux *J* of each gas and the pressure difference $\Delta P$ across the membrane is the permeance *K* of a gas in a mixture, $K=J/\Delta P$.

**Figure 3:**
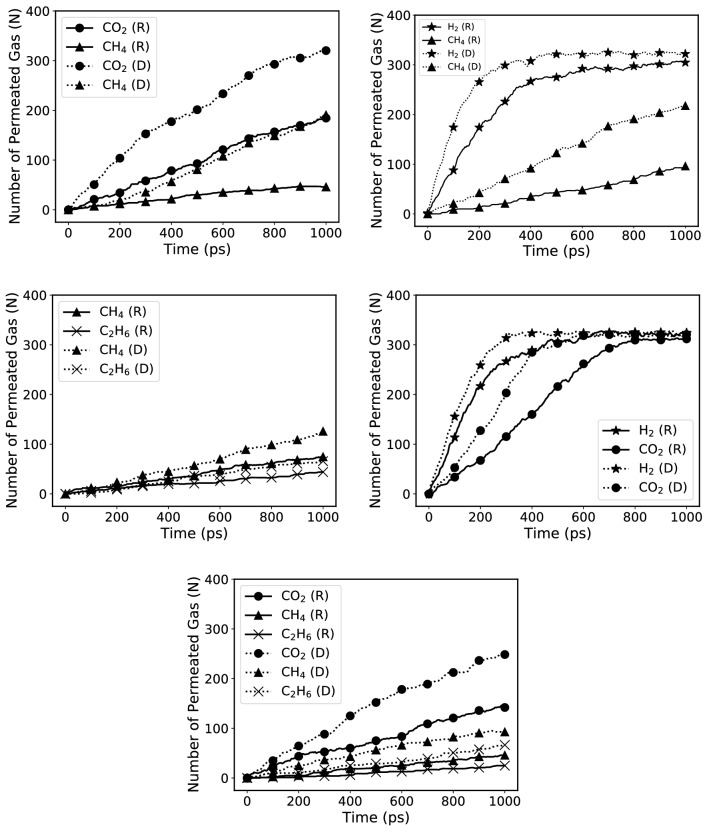
N, Number of gas molecules (H$_2$: star, CO$_2$: circle, CH$_4$: triangle, C$_2$H$_6$: cross) permeated through graphyne-3 membrane in various mixtures versus time at 300 K and a pressure difference of about 30 bars. Each mixture was simulated under the assumption that the membrane is rigid (R, solid curves) or deformable (D, dotted curves).

Figure 3 presents the dynamic evolution of the number of gas molecules permeated in both rigid and deformable membranes. Fitting a line to the first linear part of each graph yields the flux *J* for determining *K*, which is a good approximation to the actual value of the permeance in the industry, in which the flux is constant due to the large amount of the gases.

Another important property of a membrane is its selectivity or separation factor, defined as the ratio of the permeances of pairs of gases, and for the ternary mixture, we considered it as the ratio between the permeance of the desired gas and the summation of the two other gases. Table 2 summarizes estimates of the permeances and the selectivities $\alpha$. The permeances are expressed in terms of gas permeation unit (GPU), with 1 GPU representing $3.35\times 10^{-10}$ mol m$^{-2}$ s$^{-1}$ Pa$^{-1}$.

An interesting aspect of the results is that the H$_2$ permeance is about two orders of magnitude *larger* than what is produced by grephene, reported by Liu et al.^12^. The estimated permeance of $\sim 10^7$ GPU for H$_2$ is far larger than those for common membranes. This is, of course, due to the 2D structure of the membrane and the fact that the permeance is inversely proportional to the membrane thickness as mentioned before, besides the small size of H$_2$ and the strength of its interaction with graphyne-3 sheets.

The Robeson plot for CO$_2$/CH$_4$ mixtures, i.e., the selectivity of the membrane for CO$_2$ over CH$_4$ versus CO$_2$ partial pressure or permeance, was reported by Yuan et al.^60^. It is certainly important that we find the selectivity of graphyne-3 in the plot is above the Robeson upper bound, indicating that it is practically superior over all the membranes that has been studied for the separation of this mixture, including zeolites, carbon molecular-sieve membranes, and some metal-organic frameworks^61^, as well as silicon-carbide membranes.

On the other hand, the performance of graphene-3 membranes for separating the CH$_4$/C$_2$H$_6$ mixture is not superior to other types of membranes, when the selectivity is inserted in the Robeson plot. As for the ternary mixtures, the order of permeances is more or less the same as in the binary mixtures. Note the large difference between the permeance of C$_2$H$_6$ and those of CO$_2$ and CH$_4$ in the ternary mixture, implying that ethane can also be separated very efficiently from the other two gases in their mixtures.

Another important feature of Table 2 is that it points to the significance of taking into account the deformation of a membrane under the operating conditions. Under the conditions prevailing in industrial applications, a membrane is not, and cannot be assumed to be, rigid, as it will deform, which allows it to fluctuate by thermal effects. With few exceptions (see, for example, Bucior et al.^48^), however, this important point is usually ignored in computational studies of membrane separation by assuming that the membranes are rigid.

**Table 2:** Permeance (in $10^6$ GPU) and selectivity of various gas molecules in the mixtures through the rigid (R) and deformable (D) graphyne-3 membrane.

Mixture	State of the sheet	CH$_4$	CO$_2$	H$_2$	C$_2$H$_6$	Selectivity $\alpha$
CH$_4$/CO$_2$	R	0.83	2.68	–	–	(CO$_2$) 3.23
	D	2.58	8.50	–	–	(CO$_2$) 3.29
CH$_4$/H$_2$	R	1.16	–	13.05	–	(H$_2$) 11.25
	D	3.31	–	26.94	–	(H$_2$) 8.14
CH$_4$/C$_2$H$_6$	R	1.09	–	–	0.87	(CH$_4$) 1.25
	D	1.97	–	–	0.87	(CH$_4$) 2.26
CO$_2$/H$_2$	R	–	5.23	16.46	–	(H$_2$) 3.15
	D	–	8.31	20.32	–	(H$_2$) 2.45
CH$_4$/CO$_2$/C$_2$H$_6$	R	0.54	3.10	–	0.017	(CO$_2$) 5.57
	D	1.99	4.57	–	0.69	(CO$_2$) 1.71

### Effect of pressure, mixtures’ composition and system size

In the second series of MD simulations we studied the effect of pressure, mixtures’ composition, and the system size. The computations were carried out for the CO$_2$/CH$_4$ mixtures, with the expectation that qualitatively similar results should hold for the other mixtures. To be consistent with practice and for the model membrane to be more realistic, we assumed that the membrane is deformable.

**Figure 4:**
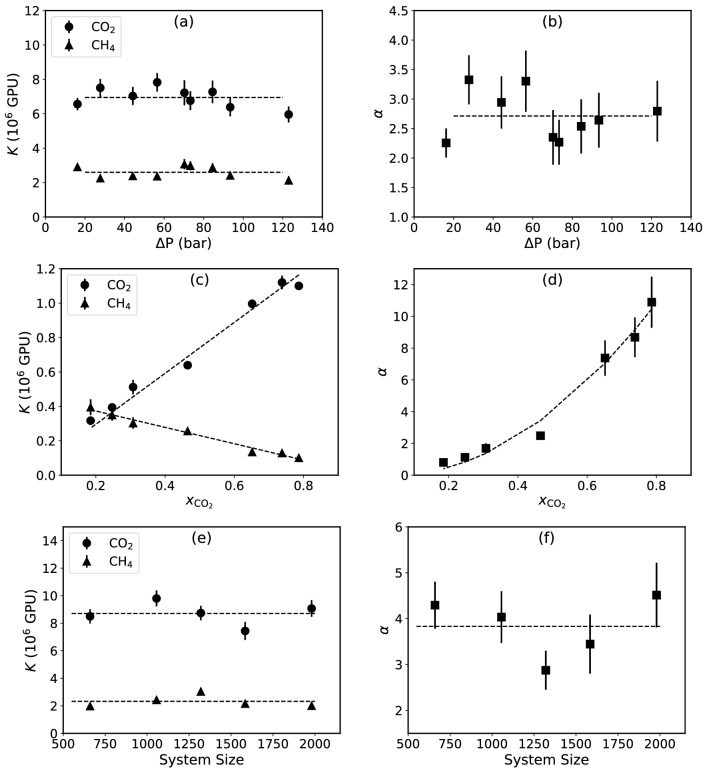
The effect of pressure (**a**,**b**), concentration (**c**,**d**) and system size (**e**,**f**) on the permeance and selectivity in the separation of CH$_4$/CO$_2$ mixture. The pressure and concentration results are for equimolar mixture with a total of 660 molecules, and the system size graphs are the results for equimolar mixtures with a 660 to about 2000 total molecules. (**a**,**b**) The permeance is independent to the pressure difference. (**c**,**d**) Increasing the concentration of CO$_2$ results in a linear increase and decrease in the permeance of CO$_2$ and CH$_4$, respectively. (**e**,**f**) Increasing the system size shows no change in the permeance and selectivity, hence showing the scalibility of the results.

To study the effect of the applied pressure *P*, we varied *P* from about 20 to 120 bar. Figure 4a, b present the results, indicating that permeances and selectivities are unaffected by *P*. Indeed, the premeances of graphyne-3 for CO$_2$ and CH$_4$ are constant and about 7.0 and 2.5 × 10^6^ GPUs, respectively.

To study the effect of the composition of the mixture, a total number of 660 molecules of CO$_2$ and CH$_4$ were used in the simulations, and the CO$_2$ number fraction ${x_{\mathrm{CO}_2}}$ was varied. Figure 4c, d present the results for the permeance and selectivity. The permeance of CO$_2$ increases linearly with ${x_{\mathrm{CO}_2}}$, whereas it decreases for the CH$_4$, which can be fitted to a 2nd-order polynomial for the selectivity $\alpha$. This is not, however, a totally surprising result, as one expects to see the linear behaviour of the permeances since the number of each gas decreases/increases in a system of constant size.

To check whether the results are independent of the number of the gas molecules used in the simulation, so that the scalability of the results can be asserted, we computed the dependence of the selectivity of the membrane for the binary mixture of CH$_4$ and CO$_2$ on the number of the gas molecules. Figure 4e, f present the results, indicating that the selectivity is independent of the number of the gas molecules. Thus, the results can be upscaled to industrial scale.

To gain a deeper understanding of the phenomena, especially the differences in the permeances of the various gases, one needs to consider the shape and size of the gases, as well as their interaction with the membrane. In the following we study at the density profiles and residence times of the gases near the membrane, and then present the results of the DFT calculations for the interaction between gases and the membrane.

### Density profiles and residence times

**Figure 5:**
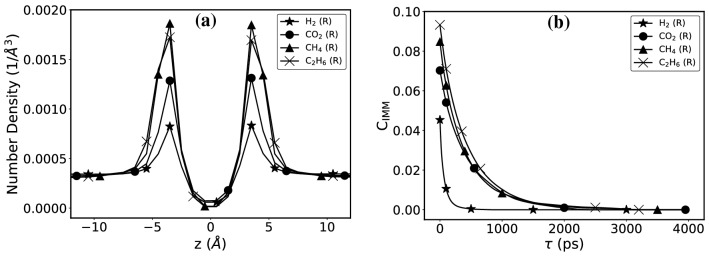
Density profiles and survival probability correlation functions of the gases near the graphyne-3 membrane, located at $z=0$, as shown in Fig. 2b. H$_2$ spends the least amount of time, compared to others, which indicates its fast transport through the membrane.

As described earlier, we simulated self-diffusion of the individual gases and their interactions with graphyne-3 in the third series of MD simulation shown in Fig. 2b. Figure 5a presents the density profiles of the four gases near the graphyne-3 sheet located in the middle of the simulation box. The peak height near the sheet indicates the number of the adsorbed molecules. Interestingly, H$_2$ is less “adsorbed” on the graphyne-3 sheet, implying that its permeance is much higher than those of the other gases. On the other side, C$_2$H$_6$ and CH$_4$ are more “adsorbed,” and CO$_2$ stands somewhere in the middle.

We also looked at how long each gas molecule reside near the surface, by computing the residence time defined, by Impey, Madden, and McDonald (IMM)^62^, who originally introduced it for quantifying how long water molecules remain near a surface in a hydration shell, which we extend to the present problem. The key quantity that IMM introduced was the *survival probability correlation function*, defined by

1 $$\begin{aligned} {C_{\rm{IMM}}} (\tau ,t^*)=\left[ \frac{\langle {p_i}(t,t+\tau ;t^*)\rangle }{\langle {p_i}(t;t^*)\rangle }\right] _{i,t}\;, \end{aligned}$$

where $p_i$ is the survival probability of molecule *i* such that, $p_i(t,t+\tau ;t^*)=1$ when the gas molecule is in the adsorption layer between time *t* and $t+\tau$ and does not leave for time intervals greater than $t^*$; otherwise, $p_i(t,t+\tau ;t^*)=0$. Here, $\langle \cdot \rangle$ represents an ensemble average over the gas molecules and time. We took $t^*$ to be 5 fs.

The results, shown in Fig. 5b, indicate that the residence times $\tau$ for C$_2$H$_6$, CH$_4$, and CO$_2$ are nearly the same, whereas ${\tau _{\mathrm{H}_2}}$ is much smaller than those of the other three gases, hence indicating that the membrane’s selectivity for H$_2$ should be very large, which is indeed the case.

Both the density profile and the residence times demonstrate the high permeance and, hence, the selctivity of graphyne-3 for H$_2$ separation. Thus, it is potentially an excellent membrane for separation of H$_2$ from other gases in a gaseous mixture. In the next section we present the results of the DFT calculations in order to assess the interaction energies of the various gases with graphyne-3, which again show hydrogen’s tendency to pass through the graphyne. In addition, we look at any possible reaction between the gases and the membrane.

### Gas-membrane interaction profiles

The number density of gas molecules and their residence time close to the nanosheet depend on their mutual interaction. To describe this interaction correctly is, therefore, critically important. We examined the interaction energy between graphyne-3 and each individual gas using the DFT calculations. The dispersion-corrected^59^ DFT energies provide a realistic description of the interaction potential energy landscape of a variety of complex systems.

**Figure 6:**
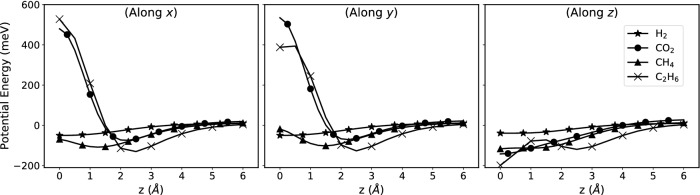
The potential energy surface of the various gases as a function of the distance from graphyne-3 membrane. The DFT method was used for the calculations. We considered three orientations of the molecule (along *x*, *y*, and *z*) and displace it along *z* direction (normal to the membrane).

We carried out the so-called single-point energy evaluations for each molecule, while it is slid rigidly and in a stepwise manner along a normal-to-the-plane axis, passing through the center of the sheet hole, as illustrated for one case in Fig. 2c. This procedure was repeated for three orientations of each molecule by aligning the main axis of the molecule either normal to the sheet plane (the *z*-direction) or parallel to it. Note that in the latter case, there are trivially two perpendicular orientations, i.e., *x*- and *y*-directions; see Fig. 2c. For example, the *z* orientation of C$_2$H$_6$ means that the C–C bond is normal to the graphyne sheet. At each separation, defined as the distance between the sheet and the molecule’s geometric center, the energy of the molecule–sheet complex was calculated. The difference between the latter and the sum of the energies of the isolated sheet and the molecule is considered as the interaction energy, which tends to zero as the separation increases, as shown for all the investigated molecules and orientations in Fig. 6.

It can be seen that the potential energy profile for hydrogen is completely different from those of the other three gases, lacking any trapping minimum, hence making it more probable than the other three gases to pass through the graphye-3 membrane. It also indicates the existence of a possible high barrier against $\hbox {C}_{2}\hbox {H}_{6}$ near the surface, hence explaining the two orders of magnitude difference between its permeance and that of $\hbox {H}_{2}$, and the smaller permeance by one order of magnitude than those of $\hbox {CO}_{2}$ and $\hbox {CH}_{4}$. Comparing $\hbox {CO}_{2}$ and $\hbox {CH}_{4}$, one can see that there exists a direction (*z*) in which both have straight paths to the membrane without barrier. The difference between this direction and the other two (*x* and *y*) is, however, much more for $\hbox {CO}_{2}$, making the *z*-orientation more preferable for $\hbox {CO}_{2}$, and giving rise to a larger permeance than $\hbox {CH}_{4}$. Therefore, the potential energy calculations also indicate the same permeance behaviour, ($\hbox {H}_{2}>\hbox {CO}_{2}>\hbox {CH}_{4}>\hbox {C}_{2}\hbox {H}_{6}$), as the MD simulations indicated. For a more assessment of the validity of using classical approach, we went further and looked at any possible interaction between the gases and graphyne-3. The next section describes the results.

### Excluding chemisorption

As the next step of validating the MD results, we focused on detecting any possible chemical reaction and chemisorption of the gas molecules with the membrane, because classical force fields that we used in the MD simulations cannot capture it. By putting a molecule initially at a series of given guessed adsorption sites, and then letting the molecule-membrane complex to relax, we checked whether they eventually develop a chemical bond at those sites. Out of about 60 test cases, only one orientation of one molecule was chemically adsorbed to the interior edge of the graphyne-3 hole. The molecule could not, however, be adsorbed as a whole, but it needed to be decomposed before chemical reaction and chemisorption can occur.

In the specific case in which chemisorption seemingly occurred, a $\hbox {C}_{2}\hbox {H}_{6}$ molecule was first dissociated into two $\hbox {CH}_{3}$ fragments, each of which could then be chemically bonded to different carbon atoms on the graphyne-3 sheet, Fig. 2d. The energy barrier of breaking the C–C bond at room temperature is too high to overcome, however, so that the reaction could occur very rarely, if at all. The adsorption energy for the exemplified case is 1.37 eV. For all the other cases examined, however, the adsorption energy was never larger than 0.25 eV, an indication of physisorption. Therefore, in the absence of accessible chemical reactions, we conclude that the classical force fields are adequate for the present MD simulations of the gas-membrane interface.

## Summary

This paper reported on the results of a study of separation of several gas mixtures containing $\hbox {CO}_{2}, \hbox {CH}_{4}, \hbox {H}_{2}$ and $\hbox {C}_{2}\hbox {H}_{6}$, using graphyne-3 as the membrane. Extensive molecular dynamics simulations and calculations based on density functional theory indicate the excellence of graphyne-3 sheets for separation of small gas molecules from the mixtures. In particular, the H$_2$ permeance through the membrane is on the order of $10^7$ GPU, by far much larger than those those in other membranes, especially in the closely-related material, graphene. The density profiles of the gases, their residence times near the graphyne-3 sheet, and their interaction energies all indicate clearly the tendency of H$_2$ to pass through the membrane, leaving C$_2$H$_6$ behind. These results, together with excellent mechanical properties of graphyne-3, confirm that the material is an excellent candidate for separating small gas molecules from gaseous mixtures, even under harsh conditions, hence opening the way for its industrial use.

## Supplementary Information

Supplementary Information 1.

## Data Availability

The datasets generated during and/or analysed during the current study are available from the corresponding author on reasonable request.
