# Supplementary material for: Graphyne-3: a highly efficient candidate for separation of small gas molecules from gaseous mixtures
Source: Sci Rep. 2021 Aug 11;11:16325. doi: 10.1038/s41598-021-95304-z (PMC8358044; doi:10.1038/s41598-021-95304-z)
Supplement: Supplementary file 1 — Supplementary Information 1. [file 41598_2021_95304_MOESM1_ESM.pdf]

SUPPLEMENTARY INFORMATION

1. Intra-molecular potential energies

For modeling the intra-molecular interactions in the gas molecules, we have used harmonic bond, harmonic angle, and the OPLS dihedral from. The energy for each type is found by:

$$E_{\text{bond}} = k_b(r - r_0)^2, \tag{1}$$

$$E_{\text{angle}} = k_a(\theta - \theta_0)^2, \tag{2}$$

$$\begin{aligned} E_{\text{dihedral}} =& \frac{1}{2}k_{d1}(1 + \cos(\phi)) + \frac{1}{2}k_{d2}(1 - \cos(2\phi)) + \\ & \frac{1}{2}k_{d3}(1 + \cos(3\phi)) + \frac{1}{2}k_{d4}(1 - \cos(4\phi)), \end{aligned} \tag{3}$$

where r, θ, and ϕ are the bond length, angle, and the dihedral angle, respectively. The parameters k_b, r_0, k_a, θ_0, k_{d1}, k_{d2}, k_{d3}, and k_{d4} are presented in Table S1 for different molecules.

Table S1: The coefficients of the bonds, angles, and the dihedral used for modeling different gas molecules, as in Equations 2-4.

Molecule	Bond	k_b (eV/Å^2)	r_0 (Å)
CH_4	C−H	14.104	1.11
CO_2	C=O	24.104	1.149
H_2	H−H	9.1	0.708
C_2H_6	C−C	11.524	1.529
	C−H	14.62	1.09

Molecule	Angle	k_a (eV)	θ_0 (degrees)
CH_4	H−C−H	3.22	109.47
CO_2	O=C=O	12.7	180
C_2H_6	C−C−H	1.6125	110.7
	H−C−H	1.419	107.8

Molecule	Dihedral	k_{d1} (eV)	k_{d2} (eV)	k_{d3} (eV)	k_{d4} (eV)
C_2H_6	H−C−C−H	0	0	0.0129	0

2. LAMMPS input file

Here we present the sample LAMMPS input file for separation of CH_4/CO_2, with a deformable graphyne-3 membrane.

```
# LAMMPS input file
boundary        p  p  p
units           metal

atom_style      full
bond_style      harmonic
angle_style     harmonic
dihedral_style  harmonic

pair_style      hybrid/overlay airebo 3.0 lj/cut/coul/cut 8.0 10

read_data       ch4_co2.data

#-------------------------------------------------------------------------

pair_coeff      1  1  lj/cut/coul/cut   0.00284   3.5     #C in CH4
pair_coeff      2  2  lj/cut/coul/cut   0.0026    2.96    #H in CH4
pair_coeff      3  3  lj/cut/coul/cut   0.00284   3.5     #C in C2H6
pair_coeff      4  4  lj/cut/coul/cut   0.0026    2.96    #H in C2H6
pair_coeff      5  5  lj/cut/coul/cut   0.006869  3.033   #C in CO2
pair_coeff      6  6  lj/cut/coul/cut   0.0024    2.757   #O in CO2
pair_coeff      7  7  lj/cut/coul/cut   0.0026    2.96    #H in H2
pair_coeff      9  9  lj/cut/coul/cut   0.0024    3.4     #C  (piston)

pair_modify     mix arithmetic

pair_coeff       1  8  lj/cut/coul/cut  0.0026107  3.45  #WRITTEN BASED ON 0.0024, 3.4
pair_coeff       2  8  lj/cut/coul/cut  0.0024980  3.18
pair_coeff       3  8  lj/cut/coul/cut  0.0026107  3.45
pair_coeff       4  8  lj/cut/coul/cut  0.0024980  3.18
pair_coeff       5  8  lj/cut/coul/cut  0.0040602  3.2165
pair_coeff       6  8  lj/cut/coul/cut  0.0024     3.0785
```

```
pair_coeff       7  8  lj/cut/coul/cut  0.0024980  3.18
pair_coeff       8  9  lj/cut/coul/cut  0.0024     3.4

pair_coeff      * * airebo CH.airebo NULL NULL NULL NULL NULL NULL NULL C
   NULL

#---------------------------------------------------------------------------

lattice          sc 3.0
region           regpiston block  0 104.937 0 96.351 118.0 120.0 units box
create_atoms     9 region regpiston

lattice   sc 3.0
region    reggycover block   0 104.937 0 96.351 -0.5 0.5 units box
create_atoms  9 region reggycover

#---------------------------------------------------------------------------

region            reg1 block 10.0 100.0  2.0   90.0  -0.5  0.5 units box
region            reg2 block 18.0 92.0   10.0  82.0  -0.5  0.5 units box

group             inreg1 region reg1
group             inreg2 region reg2
group             inregfix subtract inreg1 inreg2

group             graphyne type 8
group             gyfix intersect inregfix graphyne
group             gymobile subtract graphyne gyfix

group             piston  region regpiston
group             gycover region reggycover
group             cover subtract gycover graphyne
group             wall union cover piston
group             gas subtract all graphyne wall

group             Cch4  type 1
group             Hch4  type 2
```

```
group            Cc2h6 type 3
group            Hc2h6 type 4
group            Cco2  type 5
group            Oco2  type 6
group            Hh2   type 7

group            mobile union gymobile gas

#---------------------------------------------------------------------------

neigh_modify    exclude  group wall wall
neigh_modify    exclude  group wall graphyne

#---------------------------------------------------------------------------

velocity        gas create 300.0 12345678 dist gaussian
velocity        gyfix set 0.0 0.0 0.0
velocity        gymobile create 300.0 997899 dist uniform
velocity        wall set 0.0 0.0 0.0

#---------------------------------------------------------------------------

fix             1 gas nvt temp 300 300 0.005
fix             2 gyfix setforce 0 0 0
fix             3 wall setforce 0 0 0
fix             4 gymobile nvt temp 300 300 0.005

minimize        1.0e-4 1.0e-6 10000 100000

fix             55 gas shake 0.0001 20 0 b 3

#---------------------------------------------------------------------------

compute         temp gas temp
compute         stress all stress/atom NULL
compute         zwall wall reduce max z

```

```
variable  vol_down equal lx*ly*(c_zwall-zlo)
variable  vol_up   equal lx*ly*(zhi-c_zwall)
variable  stress   atom -(c_stress[1]+c_stress[2]+c_stress[3])/3.0
variable  press_down atom (z<c_zwall)*v_stress/v_vol_down
variable  press_up   atom (z>c_zwall)*v_stress/v_vol_up
variable  count_down atom (z<c_zwall)
variable  count_up   atom (z>c_zwall)

compute          press_down gas reduce sum v_press_down
compute          press_up   gas reduce sum v_press_up
compute          ch4_down  Cch4  reduce sum v_count_down
compute          ch4_up    Cch4  reduce sum v_count_up
compute          c2h6_down Cc2h6 reduce sum v_count_down
compute          c2h6_up   Cc2h6 reduce sum v_count_up
compute          co2_down  Cco2  reduce sum v_count_down
compute          co2_up    Cco2  reduce sum v_count_up
compute          h2_down   Hh2   reduce sum v_count_down
compute          h2_up     Hh2   reduce sum v_count_up

variable         c2h6_down equal c_c2h6_down*0.5
variable         c2h6_up   equal c_c2h6_up*0.5
variable         h2_down   equal c_h2_down*0.5
variable         h2_up     equal c_h2_up*0.5

#----------------------------------------------------------------------------

thermo_style     custom  step c_temp pe press c_press_down c_press_up c_ch4_down c_ch4_up v_c2h6_down v_c2h6_up c_co2_down c_co2_up c_h2_down c_h2_up

thermo           500
timestep         0.001
dump             1 all xyz 2000 dump1.xyz
restart          100000 restart1 restart2
run              500000

#----------------------------------------------------------------------------
```

```

unfix           3
fix             3 piston nve
fix             pullpiston piston aveforce 0.0 0.0 -0.00020

undump          1
dump            2 all xyz 2000 dump2.xyz
run             500000

#---------------------------------------------------------------------------

delete_atoms    group cover

undump          2
dump            3 all xyz 2000 dump3.xyz
run             1000000
```
